# Environmental DNA without borders: Let’s embrace decentralised genomics to meet the UN’s biodiversity targets

**DOI:** 10.1038/s44319-024-00264-w

**Published:** 2024-09-25

**Authors:** Maximilian R Stammnitz, Amber Hartman Scholz, David J Duffy

**Affiliations:** 1 Centre for Genomic Regulation, The Barcelona Institute of Science and Technology (BIST), Barcelona, Spain; 2 Leibniz Institute DSMZ, German Collection of Microorganisms and Cell Cultures, Braunschweig, Germany; 3 Whitney Laboratory for Marine Bioscience and Sea Turtle Hospital, University of Florida, St. Augustine, USA; 4 Department of Biology, College of Liberal Arts and Sciences, University of Florida, Gainesville, USA

**Keywords:** Chromatin, Transcription & Genomics, Economics, Law & Politics, Evolution & Ecology

## Abstract

Towards the 10th anniversary of the Nagoya Protocol, it is time to embrace key technology developments and adapt existing red tape for genomic monitoring.

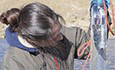

The past decade has seen a tremendous increase of genetic sequence data for biodiversity assessments and research. Third-generation DNA sequencing platforms now enable affordable, simple and reliable biomonitoring at scale (Agustinho et al, 2024; Hogg, 2024). Advances in technology have inspired large-scale international efforts, such as the *Earth BioGenome Project* (EBP) to generate high-quality references for ~1.5 million eukaryotic species (Lewin et al, 2018). In light of the ongoing sixth mass extinction, these collaborative sequencing efforts provide vital resources for ecosystem preservation and human health. Digital captures of the diversity of life on Earth support a wide range of researchers—from geneticists who aim to preserve at least the genomes of the world’s most threatened species, to ecologists who optimise conservation strategies, to epidemiologists and medical data experts who use these resources to understand pathogen evolution (Gardy and Loman, 2018).

“Third-generation DNA sequencing platforms now enable affordable, simple and reliable biomonitoring at scale.”

But it is not only research endeavours that benefit from the generation of these DNA sequences: equally important is that the resulting data are archived and made freely available to all of humanity. Nonetheless, the genomics community’s vision of globalised open-access DNA sequencing collides with existing regulatory policies on biodiversity management and ownership.

“... the genomics community’s vision of globalised open-access DNA sequencing collides with existing regulatory policies on biodiversity management and ownership.”

## Biodiversity genomics meets regulation

International frameworks that address biopiracy and illegal wildlife trade have been struggling to keep pace with the rapid digitalisation of genetics. Regulatory efforts on digital sequence information (DSI) have therefore recently led to the establishment of a new, multilateral mechanism for genetic data access and benefit sharing (Halewood et al, 2023; Salem and Kaltenpoth, 2023). Yet, one key development is frequently overlooked in this context: the progress of environmental DNA (eDNA) research.

eDNA is commonly defined as species’ genetic sequence traces in an environment, captured in the form of cell-free DNA, dead or living cells. These fragments deposit in and flow through soil, sediments, waterways and aerosols (Thomsen and Willerslev, 2015). Under the right conditions, eDNA may be stably preserved for millions of years and provide insights into ancient ecosystems (Kjær et al, 2022). At first eDNA was mainly used in microbial ecology, but nucleic acid sampling, sequencing and data handling methods have since been greatly refined for studies of macro-organismal communities as well (Blackman et al, 2024). Since the early 2010s, eDNA workflows have enabled increasingly cost-effective and routine biomonitoring. Prominent examples of worldwide eDNA surveys include the *Tara Oceans* expedition (Sunagawa et al, 2020) and the *Global Spore Sampling Project* to capture the diversity and dynamics of fungal species by air monitoring (Abrego et al, 2024) (Table 1).

**Table 1:** Large-scale biodiversity genomics and environmental DNA initiatives mentioned, in order of appearance.

Project	Aim	Webpage
Earth BioGenome Project	High-quality reference genomes of ~1.5 million eukaryotic species	https://www.earthbiogenome.org/
*Tara* Oceans expedition	Global ocean microbial biodiversity atlas via sea water eDNA capture	https://fondationtaraocean.org/en/expedition/tara-oceans/
Global Spore Sampling Project	Global fungal biodiversity atlas via air eDNA capture	https://www.helsinki.fi/en/researchgroups/spatial-food-web-ecology/research/gssp/about-gssp
*In Situ Labs*	Decentralised eDNA biomonitoring and laboratory capacity-building network with hubs in Peru, Rwanda, Vietnam and Indonesia; citizen science project	https://insitulabs.org
Cassava Virus Action Project	Mobile DNA sequencing detection of viral cassava pathogens in East Africa; citizen science project	https://cassavavirusactionproject.com
LeDNA	Global biodiversity atlas of ~800 low-altitude lakes; citizen science project	https://environmental-dna.ethz.ch/research/ercledna/global-lake-sampling.html
PyriSentinel	Cross-border biodiversity atlas of ~300 high-altitude mountain lakes; citizen science project	https://pyrisentinel.eu/
NatureMetrics	Company offering customised eDNA services	https://www.naturemetrics.com/

Geneticists are now increasingly applying PCR-free and long-read genome-sequencing methods to inspect eDNA molecules—cheaply, sensitively and remotely from their original source (Agustinho et al, 2024; Whitmore et al, 2023). These technological leaps hold new opportunities for much-needed, serial biodiversity census exercises, particularly for scientists in low- or middle-income countries. However, freely dispersing environmental substrate, the resource for eDNA analysis workflows, is challenging the premise of sovereign national rights over biodiversity use and access control, as originally foreseen under the Nagoya Protocol on Access and Benefit Sharing (NP).

“... freely dispersing environmental substrate, the resource for eDNA analysis workflows, is challenging the premise of sovereign national rights over biodiversity use and access control.”

Capture of one country’s endemic flora, fauna and microbes by eDNA monitored within the boundaries of another country is becoming an increasing reality. A number of technical and knowledge constraints do still limit the outcome for now. These include scientists’ equipment and substrate choice, uncertainties about eDNA shedding rates, dilution, decay and contamination factors (Blackman et al, 2024). However, policymakers should consider that sampling campaigns have already detected sizable eDNA fragments from distal sources. In addition to questions about equitable technology access, it may also be anticipated that eDNA transport—through both natural and anthropogenic routes—could unequally favour the strategic biodiversity monitoring position of certain countries over others.

The 10th anniversary of the NP in October 2024 coincides with the 16th Conference of the Parties (COP-16) to the UN Convention on Biological Diversity (CBD). This meeting provides an ideal occasion to align current eDNA research practices with the multilateral reform of DSI legal affairs, and with specific targets of the Kunming-Montreal Global Biodiversity Framework (GBF) adopted at COP-15 in 2022. Here, we highlight existing regulatory challenges resulting from the NP, specific trends in the eDNA community and their prospective political synergies for tackling the biodiversity crisis.

## Promises and challenges with the Nagoya Protocol

While biodiversity monitoring is a global task, historically much of the world’s DNA sequencing capacity and bioinformatics infrastructure has been concentrated in well-funded institutions in high-income countries. This mimics the global economic distribution of industrial biotechnology and its incorporation of genetic resources, and has therefore led to the adoption of major international laws that regulate in situ sampling of biodiversity.

Most prominently, access to and use of natural specimens must comply with any national laws arising from the 1992 CBD and its legally-binding supplement, the NP. The noble objective of the NP, in effect since October 2014 and since ratified by 140 states, is to encourage sustainable biodiversity management while preventing transnational economic exploitation of natural genetic resources and traditional knowledge. In theory, this is done by ensuring that the outcomes of biodiversity research and development, termed ‘benefits’, are shared with the countries of origin. In practice, implementations of the NP usually result in bilateral benefit-sharing contracts on a particular scientific project; for instance, between a ‘provider’ country with access to a species of interest and a ‘user’ research institute or company in another country.

Rather orthogonally to the NP’s legal notion of access and benefit sharing lies the broadly shared agreement in the genomics community that DSI ought to be freely accessible to researchers and the public. This consensus is mainly rooted in early efforts to contain private equity initiatives that aimed to capitalise on data from the Human Genome Project. Following from the Bermuda Accord in 1996, tax-funded human DNA sequencing data would be shared and hosted in open-access repositories (Kaye et al, 2009). The open availability of DSI has since become mainstream in genomics projects, including giant initiatives like the EBP—as long as privacy, security and traditional community rights are considered sensibly (Ebenezer et al, 2022; Sherkow et al, 2022).

It is not difficult to envision tensions between these lines of thought. The NP forms a realm in which environmental ministries, political advisers, lawyers and diplomats seek to respond to the biodiversity crisis through economic and national priorities. Biological samples are viewed fundamentally differently by those that presuppose a priori economic value from access to biodiversity versus those who see equitable long-term benefits and a common good in transnational, open-access DSI.

In practice, international biodiversity researchers often have to wait for months and navigate many bureaucratic hoops to obtain a permit under the NP. Its various implementations at the national level have led to a widely divergent, barely navigable patchwork of jurisdictions. If that Kafkaesque journey was not challenging enough, scientists may get hampered further by fine legislative differences even between regions of the same country. For example, the USA has not ratified the CBD but requires benefit-sharing agreements for biological samples from its National Parks—and there are a number of US federal eDNA-related legislative initiatives underway, potentially including new data deposition guidelines.

“In practice, international biodiversity researchers often have to wait for months and navigate many bureaucratic hoops to obtain a permit under the NP.”

**Figure 1:**
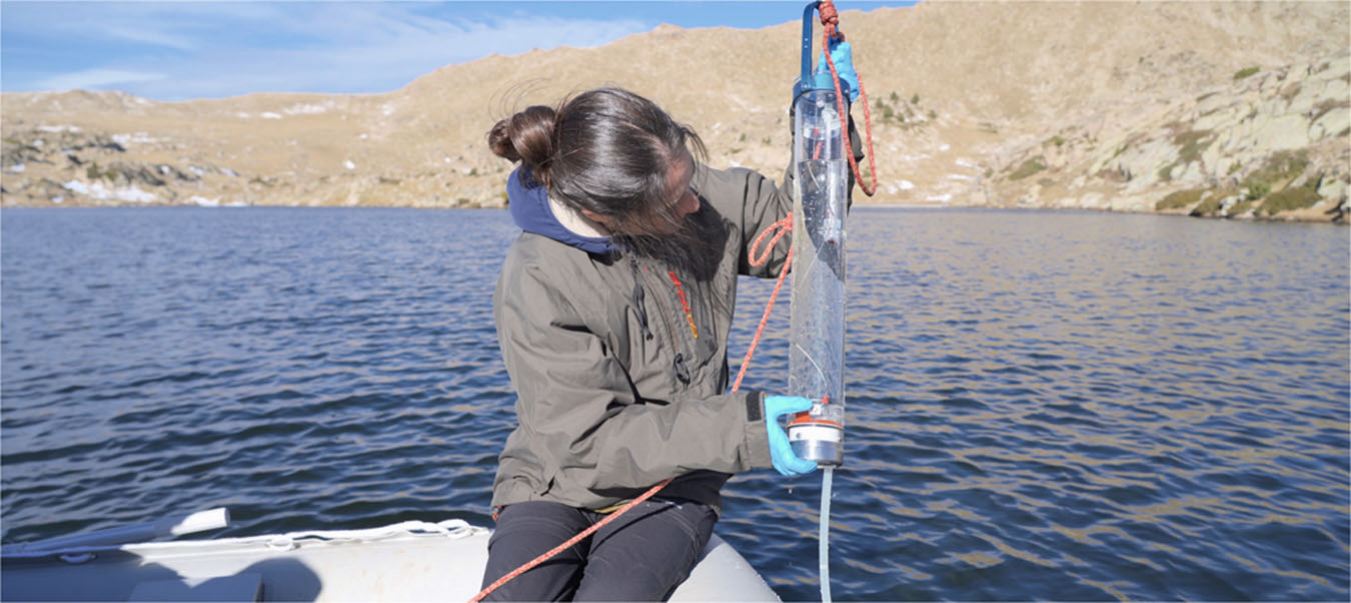
Whose eDNA? Lake water sampling in the European cross-border region of the Pyrenees. Image credit: Eduard Ros

With all these diverging legal, ethical, regional and national experiences, how should scientists and policymakers deal with the tangible subject of globally dispersing environmental substrates, as well as the organism traces and eDNA sequences carried therein? (Fig. 1) Without a doubt, the need for biodiversity protection and access limitations remains. Nonetheless, critical voices have long warned that the portrayed genetic resource evaluations of some biodiversity-rich nations and fears of biopiracy may not always be based on empirical experience—to the point at which tightened legal responses to the NP have seriously impacted their own national efforts towards conservation. This exerts a substantial strain on international research collaborations. Academics studying biodiversity in particular countries may find themselves unjustly left out because of their governments’ inefficient or indiscriminate administration of genetic resources (Deplazes-Zemp et al, 2018; Prathapan et al, 2018).

Many historic and ongoing challenges with the NP have been well-documented for the different stakeholders: Provider countries’ expectations of monetary and non-monetary benefits often remain unfulfilled, even if laws under the NP are properly implemented. Permit-handling authorities struggle to distinguish between fundamental and commercially-driven research requests. Scientists find it difficult to contact the right NP authorities, to identify and engage with the exact legal requirements for a specific permit. Non-profit organisations such as natural history museums, botanical gardens and microbial culture collections are also adversely affected by excess administration.

As DSI from biodiversity research is bound to gain further relevance over physical sample trade, a substantial decision was made at COP-15 in 2022: future DSI legislation will not be implemented under the NP, but under a new, multilateral mechanism to coordinate access to sequence data and benefit-sharing (Halewood et al, 2023; Salem and Kaltenpoth, 2023). It may prove challenging to implement this framework, yet the latest political momentum provides an opportunity to rethink and potentially simplify the NP itself.

As COP-16 in Cali, Colombia approaches, it is important to consider these global trendlines against a decade of technological progress in genomics. Members of the growing eDNA research community have learned to handle not only genetic sequencing data but also legal considerations that come with environmental substrates’ transport and natural dispersion across borders. Policymakers concerned with the development of a new biodiversity benefit-sharing mechanism under the GBF should therefore familiarise themselves with rapid developments in the eDNA sector and the associated shifts in research culture.

“As COP-16 in Cali, Colombia, approaches, it is important to consider these global trendlines against a decade of technological progress in genomics.”

## Keeping up with eDNA technology

eDNA researchers aim to refine our understanding of cross-species ecology, population structures and migration patterns (Thomsen and Willerslev, 2015; Blackman et al, 2024). Traditionally, this has been mainly carried out by capture, amplification and analyses of short taxonomic marker sequences such as the variable regions of ribosomal RNA genes—approaches termed ‘metabarcoding’. Owing to the broad simplification and cost reduction of long-read DNA sequencing, however, scientists are now rapidly improving the resolution of whole-genome sequencing from environmental substrates (Agustinho et al, 2024). This translates into several additional advantages over metabarcoding, for example by enhancing inferences of species’ genetic diversity and adaptive potential. Applied to complex DNA samples, these long-read sequencing techniques can already be used to track individual species’ subpopulation structure, estimate their geographical origins and detect rare chromosomal deletion polymorphisms (Whitmore et al, 2023). In the future, ‘adaptive’ sequencing strategies may even allow for the enrichment of rare, cryptic or hitherto unknown species’ DSI traces from diverse micro- and macro-organismal communities.

The increase and diversification of eDNA applications creates new legal challenges, such as forensics and ancestry profiling opportunities which conflict with privacy and surveillance ethics. Concerns about informative human ‘bycatch’ sequences in environmental isolates have recently triggered a bioethics debate (Whitmore et al, 2023). Novel eDNA enrichment and genome-sequencing methods are tilting the scales of historic incoherencies of the NP, too. For instance, it has long been known that bacteria can disperse widely and make it difficult for microbiologists to unequivocally pinpoint their geographical origin (Salem and Kaltenpoth, 2023). Improvements to eDNA sequencing are now bound to recapitulate the same dilemma for higher organisms—surveys of airborne eDNA have already resolved the combined profiles of not only microbial communities but also diverse fungi, plants, insects, birds and even terrestrial mammals (Abrego et al, 2024; Littlefair et al, 2023).

This makes for a compelling case to consider many eDNA samples as transboundary by default, and for these to be governed multilaterally. Policymakers should tap into experiences with the UN Biodiversity Beyond National Jurisdiction (‘High Seas’) Treaty, which was recently adopted after nearly two decades of negotiation. Here, in order to assure the sustainable and fair economic use of marine genetic resources derived from international waters, a number of multilateral solutions and obligations are currently being considered (Halewood et al, 2023). These include commercialisation revenue payments from biotechnology companies into a common fund, or non-monetary benefits such as open DSI sharing with public third parties.

## Portability, citizen science and decentralisation

Up until the early days of the NP, most high-throughput DNA sequencing machines were the size of a small refrigerator, cost upwards of US$100,000, and needed to be operated and maintained by specialists. 2014 then saw the commercial appearance of Oxford Nanopore Technologies’ MinION, the world’s smallest long-read DNA sequencing device which can be powered and operated by a standard laptop (Agustinho et al, 2024). Its low cost, portability and much simplified protocol handling have since transcended the field of genomics. Deployments of MinION sequencers have already played crucial roles in remote eDNA surveys of polar, mountain or rainforest locations, and in real-time tracings of viral outbreaks around the globe (Gardy and Loman, 2018).

Clearly, the funding, logistics and human expertise required for bespoke sequencing campaigns are steadily decreasing. Already well illustrated by international initiatives like *In Situ Labs*, future eDNA biodiversity screens may be undertaken by smaller, decentralised groups or individual researchers (Gardy and Loman, 2018; Watsa et al, 2020). With this simplification comes a welcome shift of the traditional boundaries of the scientific community; indeed, academic leaders in genomics are increasingly aware of the added value of citizen participation. As a prime example, the *Cassava Virus Action Project* works closely with farmers in Tanzania, Uganda and Kenya to monitor devastating crop pathogens by MinION sequencing (Boykin et al, 2019). Both recently launched international *LeDNA* and *PyriSentinel* initiatives aim to carry out eDNA biodiversity assessments of hundreds of low- and high-altitude lakes, respectively, with water samples collected by professional researchers, park rangers and volunteers. The EBP emphasises the boost it expects from the involvement of students and hobby practitioners (Lewin et al, 2018). As illustrated by the successes of enterprises such as *NatureMetrics*, there is also a growing commercial scene in which collaborative eDNA solutions are sought for various stakeholders, from local conservationists to project leads at the national level (Table 1).

While the benefits to decentralised community science have been long recognised in traditional ecological surveys such as bird counts, this momentum is still young with respect to eDNA biomonitoring. Portable sequencing devices act as catalysts to this end—in the right settings, citizens may collect and sequence their own environmental samples to obtain fast, personalised insights. It is also a current overarching theme in genomics to enable undergraduate students and even laboratory novices to perform the basic workflows of eDNA taxonomy (Watsa et al, 2020).

These shifts in research practice have further implications for the bilateral premises of the NP: nations with less financial leeway and longer travel distances will welcome the prospect of self-assessing their own biodiversity. Mobile sequencing comes with a reduction in costly shipping of genetic resources to facilities abroad and, consequently, fewer roadblocks around permits and the all-too-common lack of acknowledgement for local scientists and field workers in research publications (Ebenezer et al, 2022). New ‘lab in a suitcase’ approaches to eDNA monitoring pose a tempting alternative to traditional user countries as well—rather than having to undergo lengthy negotiations on sample imports, it has become more straightforward for their scientists to travel to distal biodiversity hotspots and generate DSI on the ground. A global reduction in physical sample trade volume should be appreciated by legislators, though the diversification of sequencing capacity comes with the challenge of loosened transparency on the sources of genetic data.

## A call for reasonable governance on eDNA research

DSI and eDNA monitoring are, in numerous ways, essential and co-dependent for the implementation of the GBF 2030 targets. The future multilateral DSI access and benefit-sharing mechanism should therefore recognise the need to carefully harmonise digital and physical sampling spheres. For instance, eDNA researchers and citizens with portable, non-invasive sequencing equipment can play a vital role in implementing GBF Target 4 to “maintain and restore genetic diversity within and between populations of native, wild and domesticated species to maintain their adaptive potential”. However, the positive leverage of powerful genomics solutions and their widespread application is only maximised if resulting data are generated, shared and accessed without overly restrictive administrative terms.

“DSI and eDNA monitoring are, in numerous ways, essential and co-dependent for the implementation of the GBF 2030 targets.”

On its 10th anniversary at COP-16, the NP is due for a crucial re-evaluation. Constructive assessments of the past decade’s access and benefit-sharing practice need to acknowledge the ongoing digitalisation of genetics, as new technology trends are rapidly conquering research. Altogether, this asks for a simplified response to the generation and exchange of eDNA data.

We are at the dawn of a new era of data mining in biology. Artificial intelligence models are already trained on hundreds of millions of genetic sequences from across the tree of life, improving human medicine and our fundamental understanding of evolution. Countless environmental samples collected and sequenced by researchers throughout our planet’s biosphere underpin this. Ultimately, DNA is the ‘magic’ molecule at the core of these efforts. It travels through oceans, rivers, clouds and subways. It has no nationality. It knows no borders.

## Supplementary information

Peer Review File
