# Supplementary material for: Environmental DNA without borders: Let’s embrace decentralised genomics to meet the UN’s biodiversity targets
Source: EMBO Rep. 2024 Sep 25;25(10):4095–9. doi: 10.1038/s44319-024-00264-w (PMC11467388; doi:10.1038/s44319-024-00264-w)
Supplement: Supplementary file 1 — Peer Review File [file 44319_2024_264_MOESM1_ESM.pdf]

EMBO reports

Environmental DNA without borders

Maximilian Stammnitz, Amber Hartman Scholz, and David Duffy

Corresponding author(s): Maximilian Stammnitz (maximilian.stammnitz@crg.eu)

**Review Timeline:**	Submission Date:	6th Aug 24
	Accepted:	11th Sep 24

Editor: Holger Breithaupt

Transaction Report: This article was editorially reviewed.
